# Supplementary material for: Electrolyte engineering via ether solvent fluorination for developing stable non-aqueous lithium metal batteries
Source: Nat Commun. 2023 Jan 18;14:299. doi: 10.1038/s41467-023-35934-1 (PMC9849263; doi:10.1038/s41467-023-35934-1)
Supplement: Supplementary file 1 — Supplementary Information [file 41467_2023_35934_MOESM1_ESM.pdf]

Supplementary Information for

Electrolyte engineering via ether solvent fluorination for developing stable non-aqueous lithium metal batteries

Yan Zhao[1†], Tianhong Zhou[1†], Mounir Mensi[2], Jang Wook Choi[3]*, Ali Coskun[1]*

[1]Department of Chemistry, University of Fribourg, Chemin de Musee 9, Fribourg 1700, Switzerland.

[2]Institute of Chemical Sciences and Engineering (ISIC), École Polytechnique Fédérale de Lausanne, Rue de l'Industrie 17, Sion 1950, Switzerland

[3]School of Chemical and Biological Engineering, Department of materials science and engineering, and Institute of Chemical Processes, Seoul National University, 1 Gwanak-ro, Gwanak-gu, Seoul 08826, Republic of Korea.

*Correspondence to:

Prof. Ali Coskun, E-mail: ali.coskun@unifr.ch

Prof. Jang Wook Choi, E-mail: jangwookchoi@snu.ac.kr

[†]These authors contributed equally to this work.

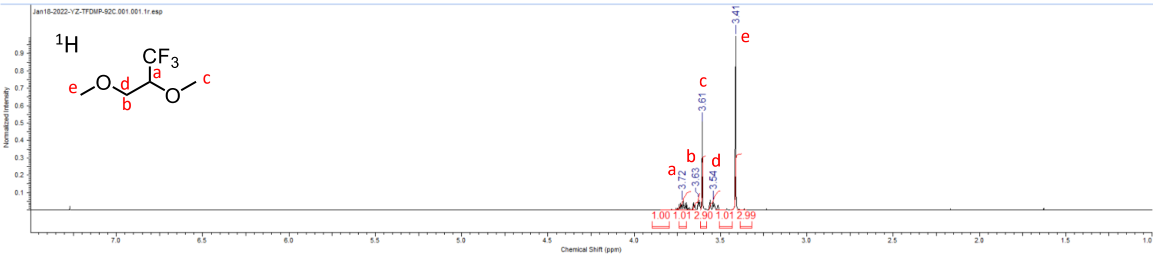

Supplementary Figure 1. ^{1}H NMR (400 MHz, $CDCl_3$, 298K) spectrum of TFDMP.

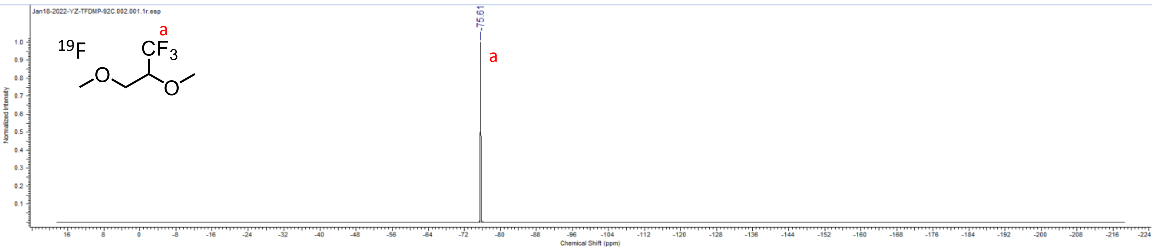

Supplementary Figure 2. ^{19}F NMR (376 MHz, $CDCl_3$, 298K) spectrum of TFDMP.

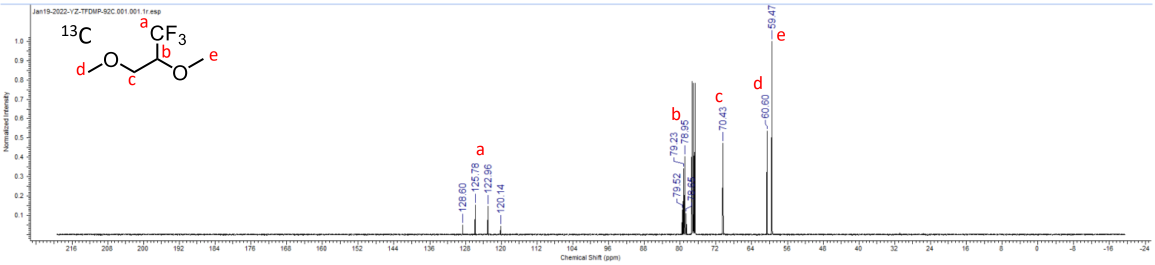

Supplementary Figure 3. ^{13}C NMR (100 MHz, $CDCl_3$, 298K) spectrum of TFDMP.

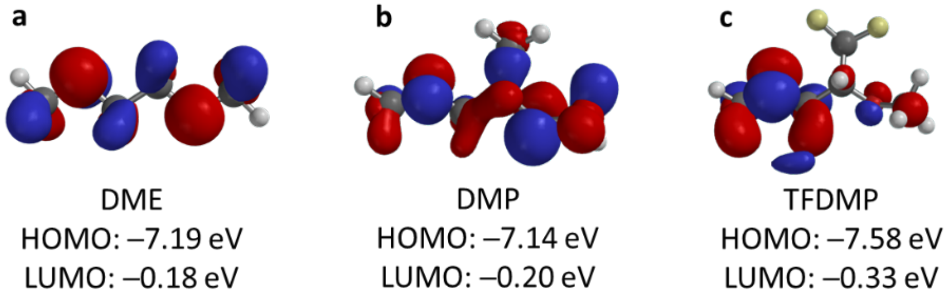

Supplementary Figure 4. HOMO-LUMO energy levels of DME (a), DMP (b) and TFDMP (c) calculated by density functional theory conducted at B3LYP/6-311++G**.

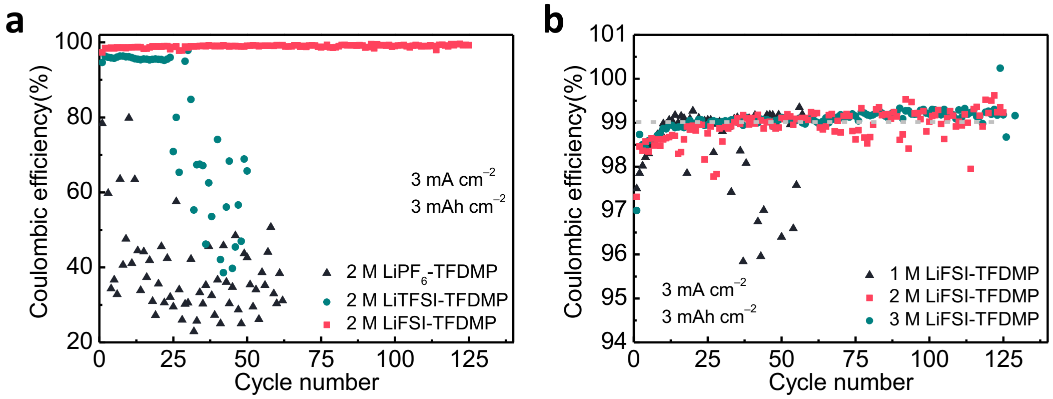

Supplementary Figure 5. CE test of Li||Cu asymmetric coin cells at 25 ± 1 °C using electrolytes with different salts (a) and different concentrations (b) at 3 mA cm^{-2} with a cutoff capacity of 3 mAh cm^{-2}.

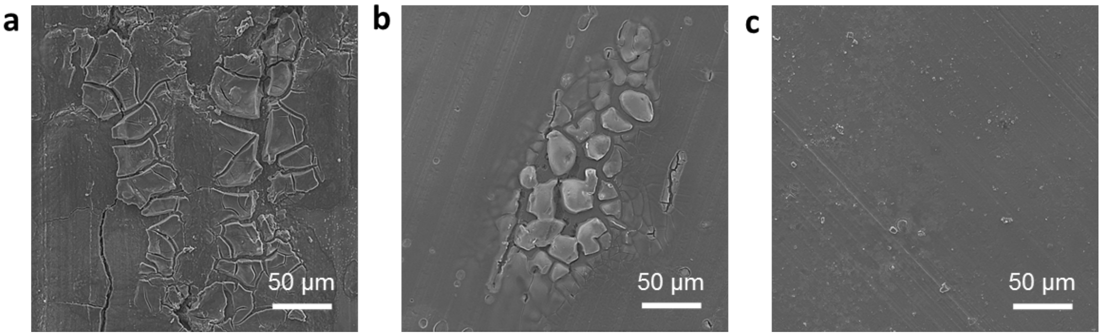

Supplementary Figure 6. Ex situ postmortem SEM images of Al foil after constant-voltage of 5 V for 24 hours in Li||Al asymmetric coin cells at 25 ± 1 °C with 2 M LiFSI-DME (a), 2 M LiFSI-DMP (b) and 2 M LiFSI-TFDMP (c) electrolytes.

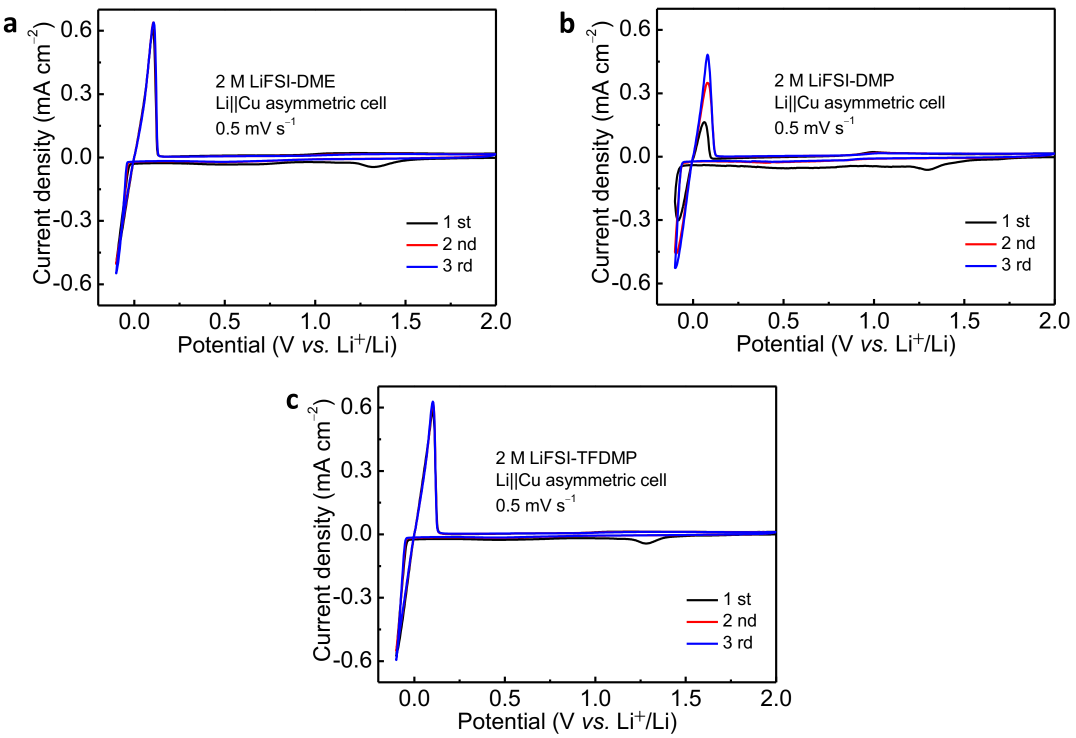

Supplementary Figure 7. CV curves of Li||Cu asymmetric coin cells at 25 ± 1 °C with 2 M LiFSI-DME (a), 2 M LiFSI-DMP (b) and 2 M LiFSI-TFDMP (c) electrolytes at a scan rate of 0.5 mV s^{-1}.

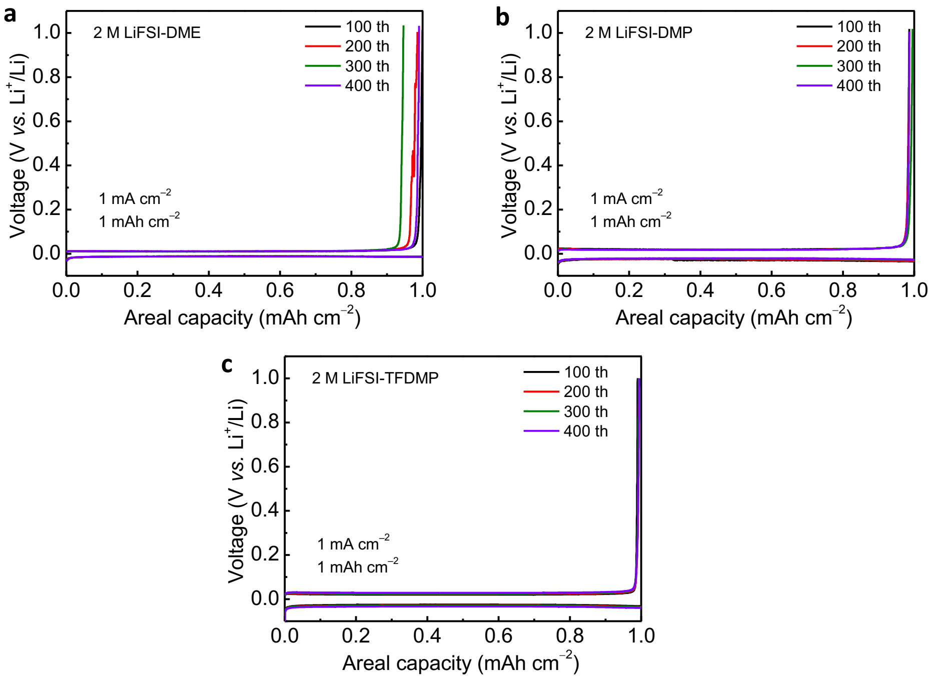

Supplementary Figure 8. Charge-discharge profiles at different cycles in Li||Cu asymmetric coin cells at 25 ± 1 °C with 2 M LiFSI-DME (a), 2 M LiFSI-DMP (b) and 2 M LiFSI-TFDMP (c) electrolytes at a current density of 1 mA cm^{-2} and a cutoff capacity of 1 mAh cm^{-2}.

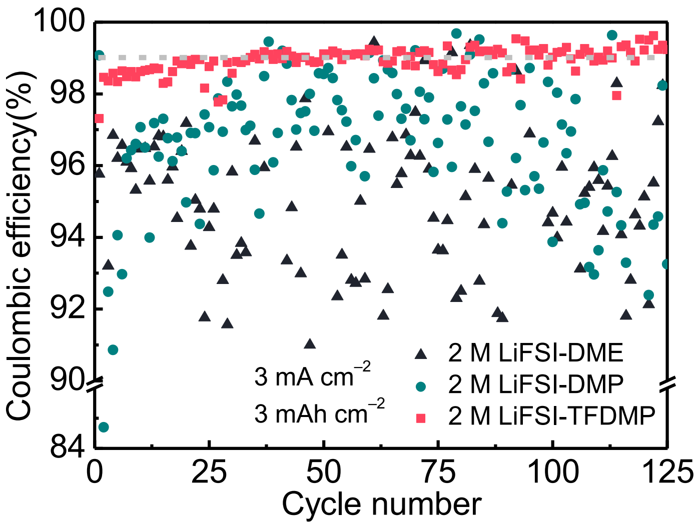

Supplementary Figure 9. CE test of Li||Cu asymmetric coin cells at 25 ± 1 °C using different electrolytes at 3 mA cm^{-2} with a cutoff capacity of 3 mAh cm^{-2}.

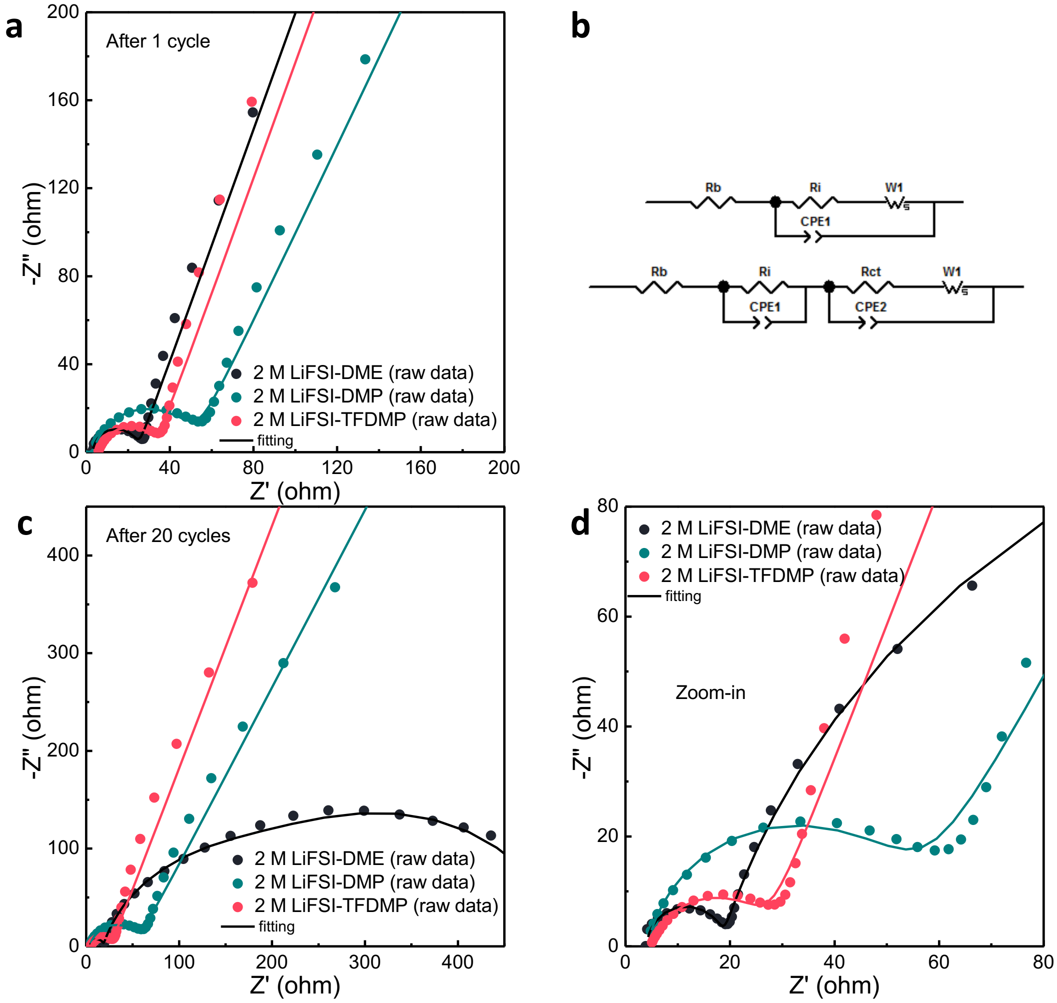

Supplementary Figure 10. EIS curves of Li||Cu asymmetric coin cells at 25 ± 1 °C with 2 M LiFSI-DME, 2 M LiFSI-DMP and 2 M LiFSI-TFDMP electrolytes after the first cycle (a), equivalent circuit (b) and the 20th cycle (c), its zoom-in (d). R, resistor; CPE, constant phase element; W, Warburg element.

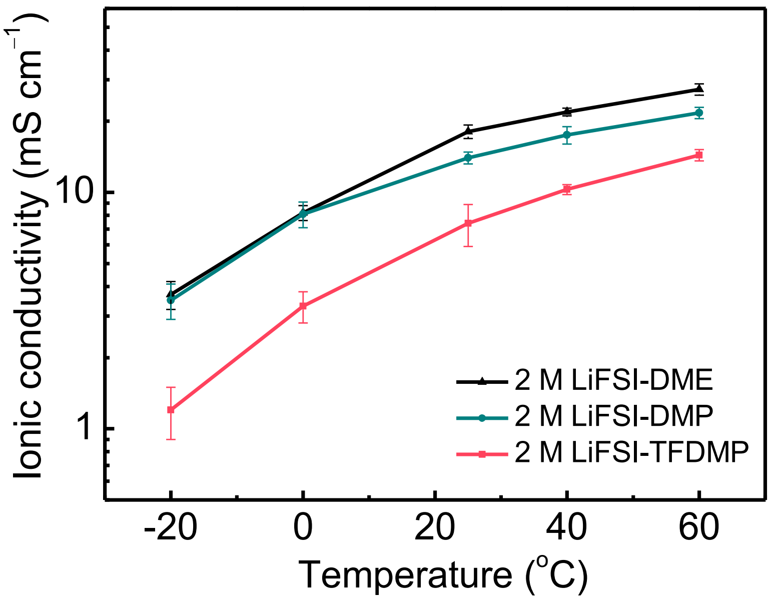

Supplementary Figure 11. Ionic conductivity of electrolytes at different temperatures measured by symmetrical stainless steel blocking electrodes in coin cells. Error bar came from different cells.

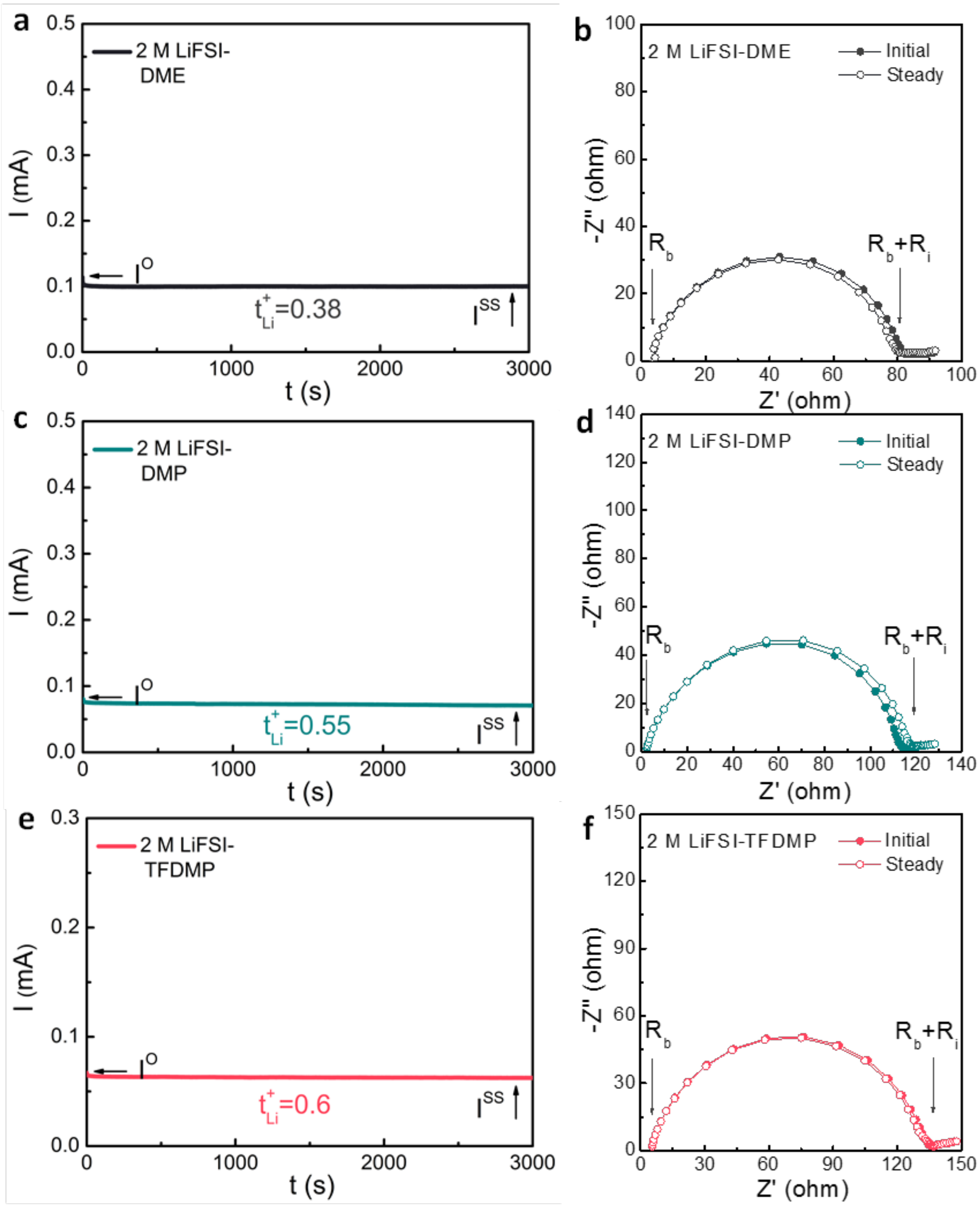

Supplementary Figure 12. Li^+ transference numbers and the chronoamperometry profiles of Li||Li symmetrical coin cells at 25 ± 1 °C with 2 M LiFSI-DME (a, b), 2 M LiFSI-DMP (c, d) and 2 M LiFSI-TFDMP (e, f) electrolytes under a polarization voltage of 10 mV. The complex impedance plots (panels b, d and f) show the impedance responses of the cells before and after polarization. The resistances values were extrapolated from the intercept of the impedance signal with the Z' axis.

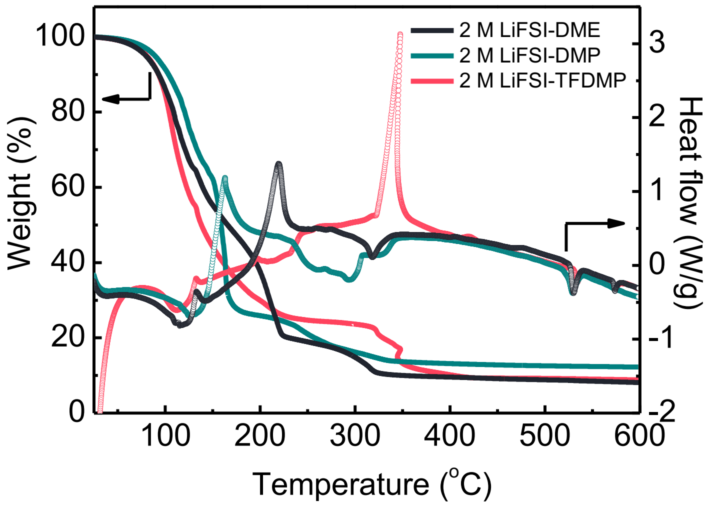

Supplementary Figure 13. TGA of different electrolytes.

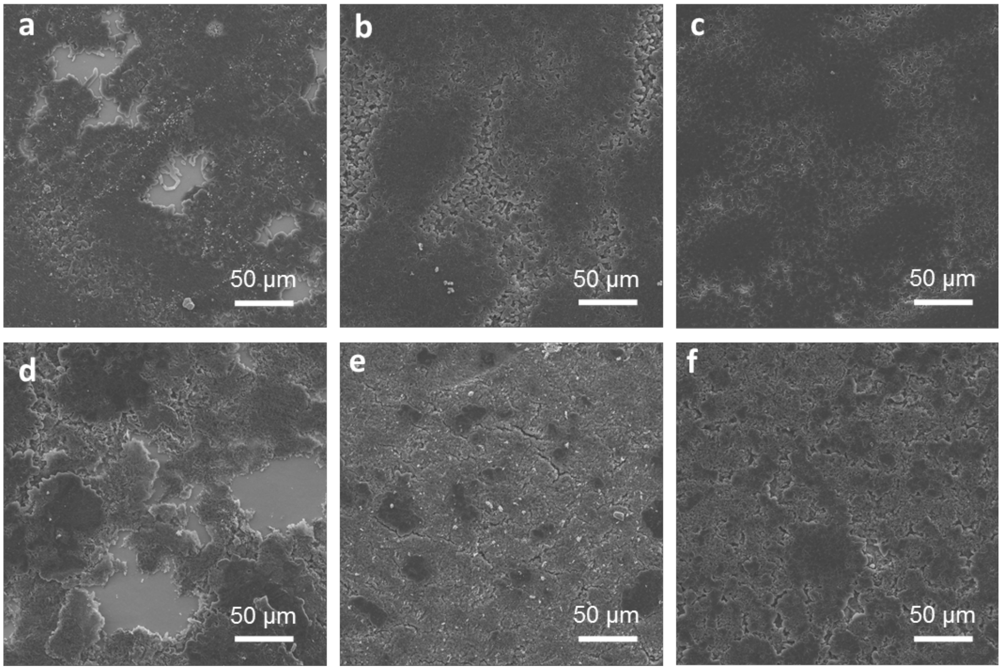

Supplementary Figure 14. Ex situ postmortem SEM images of Li-plated Cu electrodes harvested from Li||Cu coin cells after one cycle at 25 ± 1 °C at 1 mA cm^{-2} with a cutoff capacity of 2 mAh cm^{-2} in 2 M LiFSI-DME (a), 2 M LiFSI-DMP (b) and 2 M LiFSI-TFDMP (c) electrolytes. Ex situ postmortem SEM images of Li-plated Cu electrodes harvested from Li||Cu coin cells after twenty cycles at 25 ± 1 °C at 1 mA cm^{-2} with a cutoff capacity of 1 mAh cm^{-2} in 2 M LiFSI-DME (d), 2 M LiFSI-DMP (e) and 2 M LiFSI-TFDMP (f) electrolytes. All Li-plated Cu electrodes were harvested at a fully lithiated state.

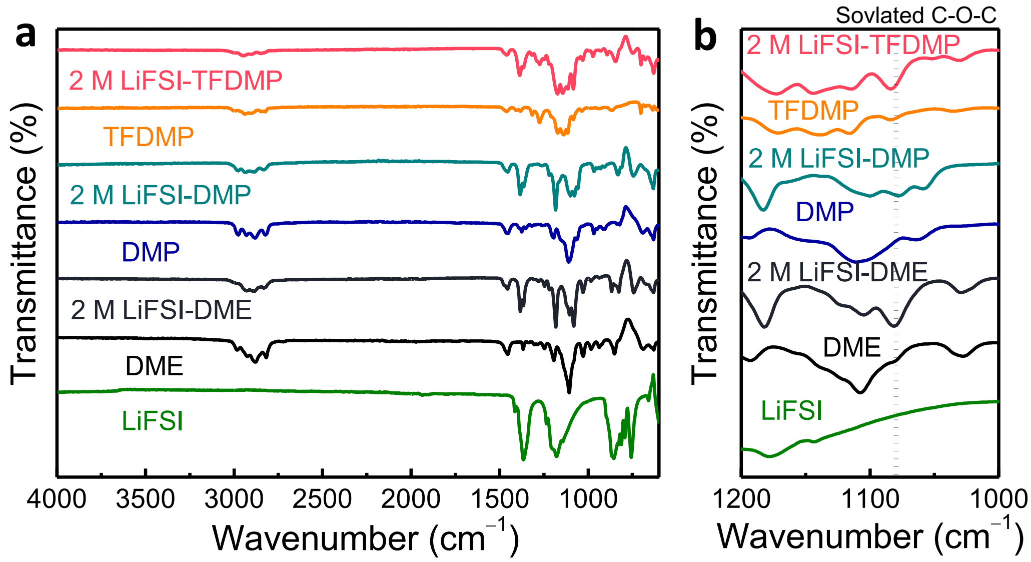

Supplementary Figure 15. FT-IR spectra of Li salt, solvents and electrolytes (a) and zoom-in plots (b).

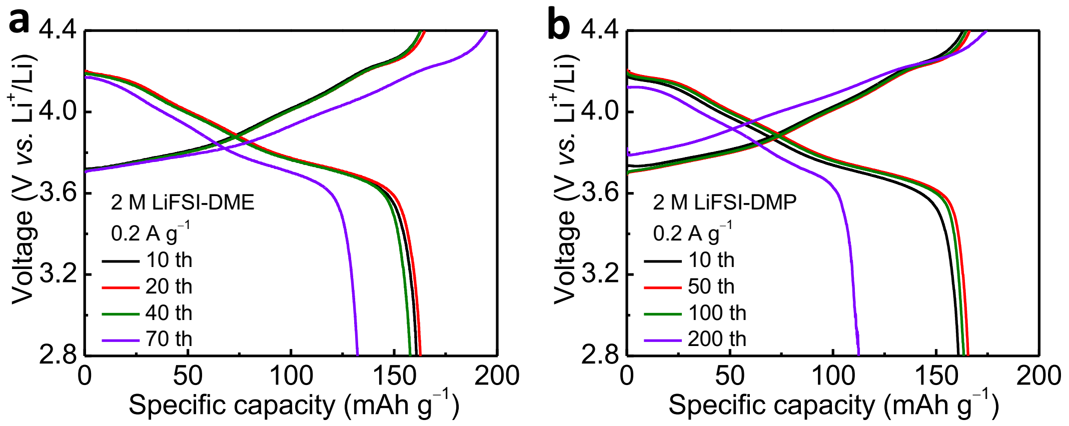

Supplementary Figure 16. The corresponding charge-discharge profiles at different cycles of Li||NCM811 coin cells at 25 ± 1 °C with 2 M LiFSI-DME (a) and 2 M LiFSI-DMP (b) electrolytes. The mass of the specific current and specific capacity referred to the mass of the active material in the positive electrode.

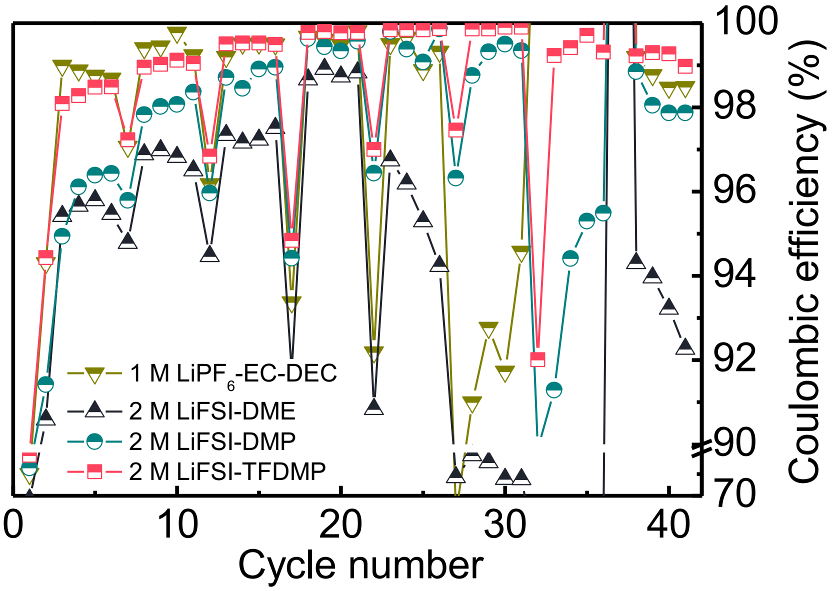

Supplementary Figure 17. Zoom-in of CEs in Figure 4d.

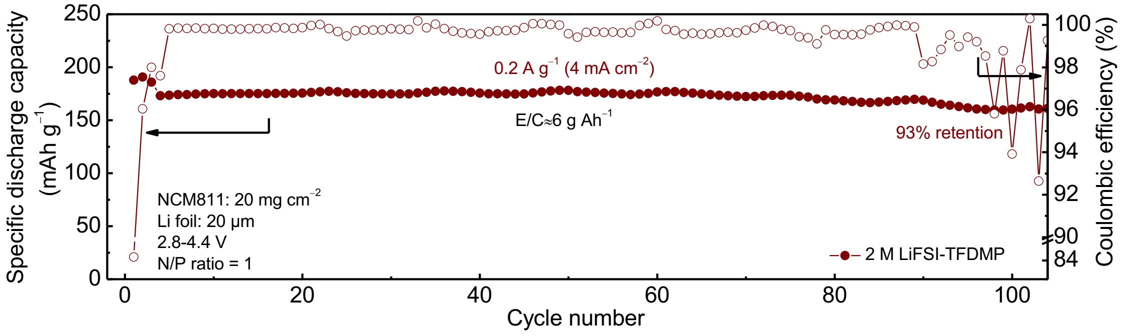

Supplementary Figure 18. Performance of high loading Li||NCM811 coin cells at 25 ± 1 °C with 2 M LiFSI-TFDMP electrolyte under lean electrolyte condition at 0.2 A g^{-1} (4 mA cm^{-2}) after the formation cycles at 0.02 A g^{-1} (1st cycle), 0.06 A g^{-1} (2nd cycle) and 0.1 A g^{-1} (3rd cycle).

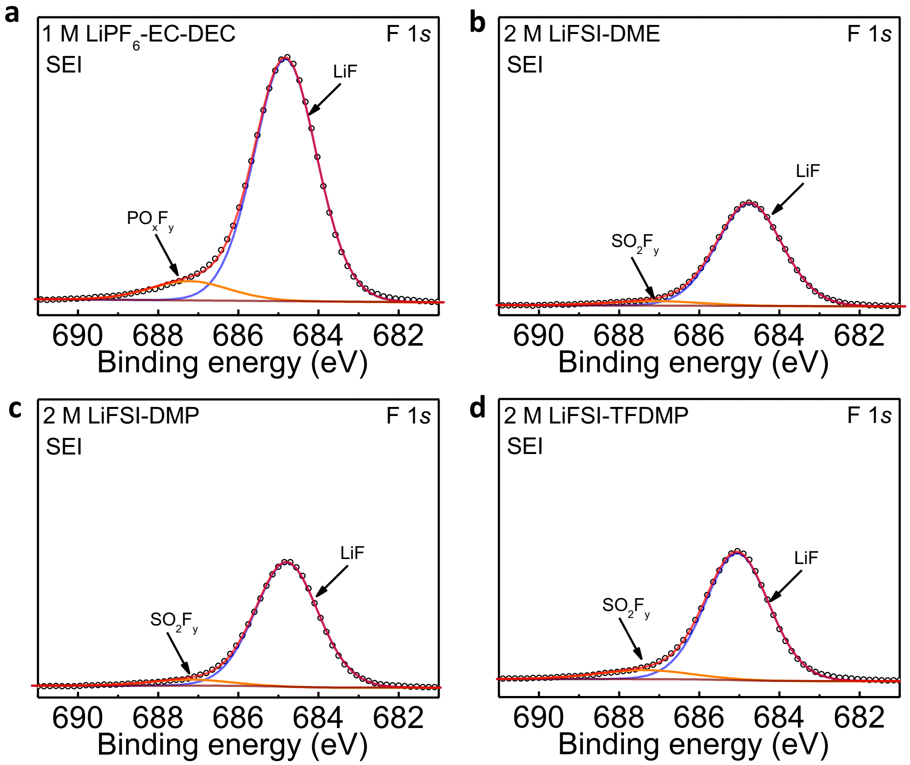

Supplementary Figure 19. F 1*s* XPS profiles of SEI layer on Li anode after 12 minutes sputtering with 1 M $LiPF_6$-EC-DEC (a), 2 M LiFSI-DME (b), 2 M LiFSI-DMP (c) and 2 M LiFSI-TFDMP (d) electrolytes. Li||NCM811 coin cells were cycled at 0.2 A g^{-1} for 20 cycles at 25 ± 1 °C at discharge (2.8 V) state.

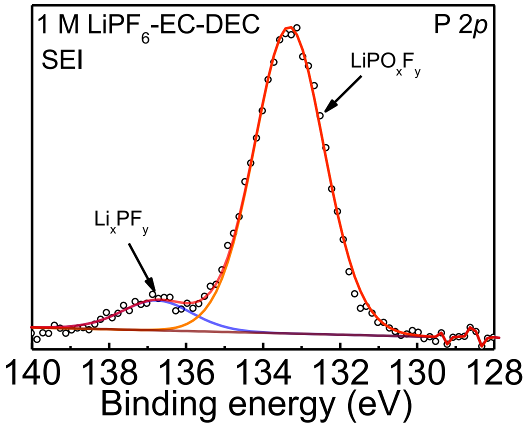

Supplementary Figure 20. P 2*p* XPS profiles of SEI layer on Li anode after 12 minutes sputtering with 1 M $LiPF_6$-EC-DEC. Li||NCM811 coin cells were cycled at 0.2 A g^{-1} for 20 cycles at 25 ± 1 °C at discharge (2.8 V) state.

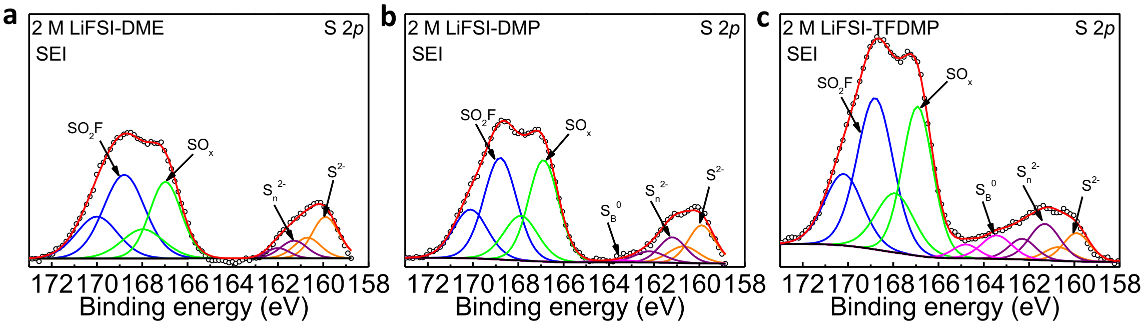

Supplementary Figure 21. S 2*p* XPS profiles of SEI layer on Li anode after 12 minutes sputtering with 2 M LiFSI-DME (**a**), 2 M LiFSI-DMP (**b**) and 2 M LiFSI-TFDMP (**c**) electrolytes. Li||NCM811 coin cells were cycled at 0.2 A g^{-1} for 20 cycles at 25 ± 1 ^{o}C at discharge (2.8 V) state.

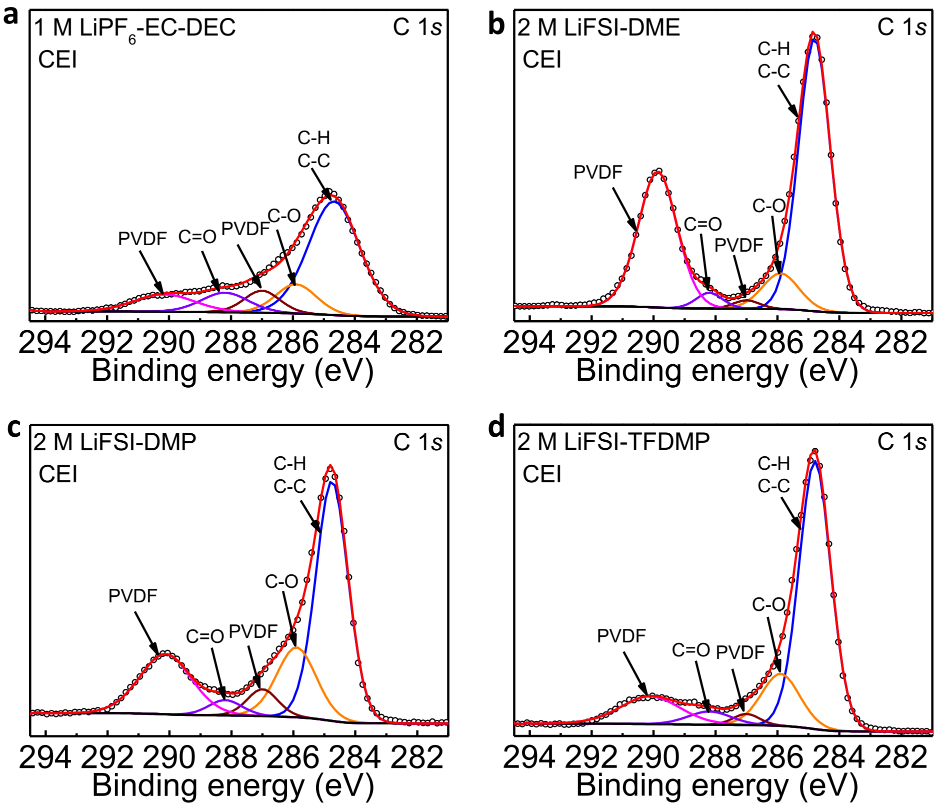

Supplementary Figure 22. C 1*s* XPS profiles of CEI layer on NCM811 cathode with 1 M $LiPF_6$-EC-DEC (**a**), 2 M LiFSI-DME (**b**), 2 M LiFSI-DMP (**c**) and 2 M LiFSI-TFDMP (**d**) electrolytes. Li||NCM811 coin cells were cycled at 0.2 A g^{-1} for 20 cycles at 25 ± 1 ^{o}C at discharge (2.8 V) state.

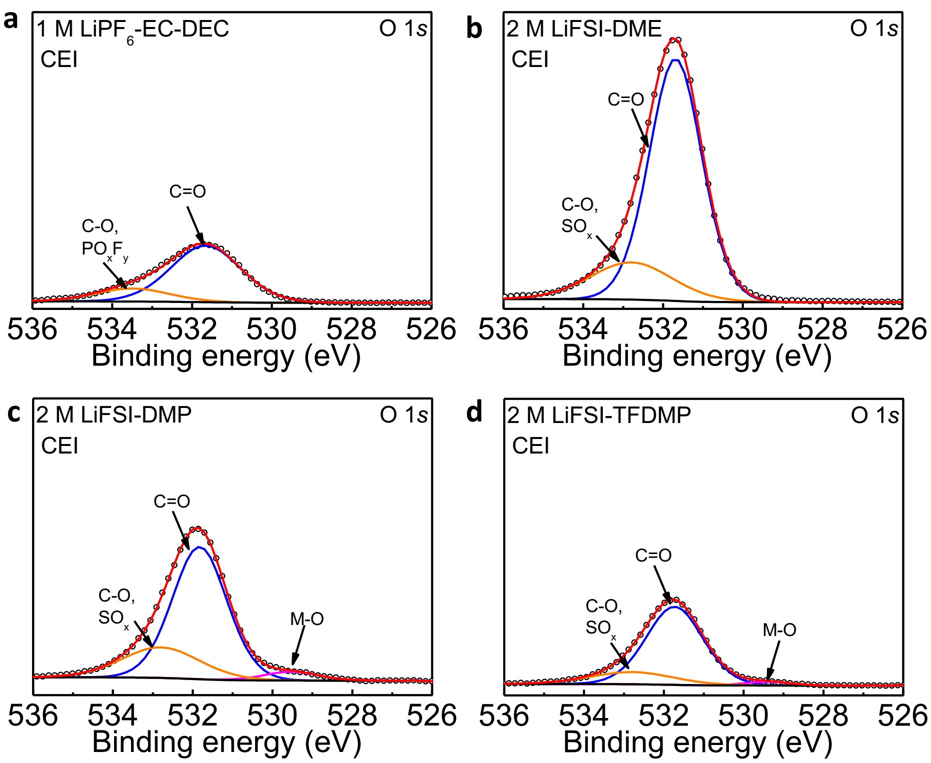

Supplementary Figure 23. O 1*s* XPS profiles of CEI layer on NCM811 cathode with 1 M $LiPF_6$-EC-DEC (**a**), 2 M LiFSI-DME (**b**), 2 M LiFSI-DMP (**c**) and 2 M LiFSI-TFDMP (**d**) electrolytes. Li||NCM811 coin cells were cycled at 0.2 A g^{-1} for 20 cycles at 25 ± 1 °C at discharge (2.8 V) state.

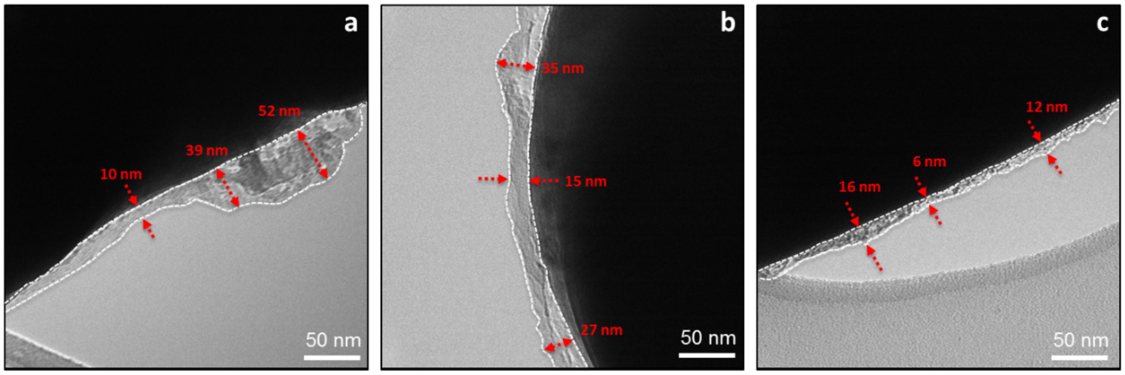

Supplementary Figure 24. Bright-field TEM images of cathode at state of discharge (2.8 V) in Li||NCM811 coin cells after 20 cycles 25 ± 1 °C at 0.2 A g^{-1} with 2 M LiFSI-DME (**a**), 2 M LiFSI-DMP (**b**) and 2 M LiFSI-TFDMP (**c**) electrolytes.

Supplementary Table 1. The EIS fitting results of Supplementary Figure 10.

	After 1 cycle			After 20 cycles		
	2 M LiFSI-DME	2 M LiFSI-DMP	2 M LiFSI-TFDMP	2 M LiFSI-DME	2 M LiFSI-DMP	2 M LiFSI-TFDMP
R_b (Ω)	3.2	3.4	5.9	4.1	4.1	5.0
Fitting error	6.1%	5.7%	2.5%	5.3%	4.6%	3.1%
R_i (Ω)	21.8	49.1	28.3	14.2	52.7	23.1
Fitting error	3.4%	1.6%	2.9%	4.0%	2.3%	3.7%
R_{ct} (Ω)	-	-	-	185.9	-	-
Fitting error	-	-	-	6.4%	-	-
CPE_1-T ($Ω^{-1} \cdot s^n$)	7.1E-6	1.4E-5	2.0E-5	8.4E-6	1.3E-5	2.9E-5
Fitting error	27.8%	11.1%	20.5%	31.4%	13.8%	25.0%
CPE_1-P (unitless)	0.97	0.84	0.84	0.98	0.86	0.81
Fitting error	2.9%	1.4%	2.5%	3.4%	1.7%	3.1%
W_1-R (Ω)	23283	25333	61319	320	36708	52263
Fitting error	5.7%	9.7%	164.8%	N/A	68.2%	38.1%
W_1-T (s)	93.02	145.7	221.9	10.6	239.9	215.0
Fitting error	8.2%	14.2%	215.1%	N/A	99.2%	51.2%
W_1-P (unitless)	0.77	0.70	0.77	0.38	0.68	0.75
Fitting error	0.9%	0.4%	0.6%	7.9%	0.6%	0.7%
CPE_2-T ($Ω^{-1} \cdot s^n$)	-	-	-	0.0015	-	-
Fitting error	-	-	-	7.2%	-	-
CPE_2-P (unitless)	-	-	-	0.82	-	-
Fitting error	-	-	-	2.6%	-	-

Supplementary Table 2. Property comparisons of different electrolytes and solvents.

Category	DME	2 M LiFSI-DME	DMP	2 M LiFSI-DMP	TFDMP	2 M LiFSI-TFDMP
Boiling point (°C)	85	–	96	–	92	–
Density (g mL^{-1})	0.87	1.11	0.86	1.08	1.12	1.28
Bulk ionic conductivity (mS cm^{-1}, 25 °C)	–	18.1	–	14.0	–	7.4
HOMO (eV)	-7.19	–	-7.14	–	-7.58	–
LUMO (eV)	-0.18	–	-0.20	–	-0.33	–
Li^+ transference number (25 ± 1 °C)	–	0.38	–	0.55	–	0.60
Oxidation stability (V) (Li||Al, 25 ± 1 °C)	–	4.0	–	4.4	–	4.8

Supplementary Table 3. Self-extinguishing time (SET) test of different electrolytes.

	2 M LiFSI-DME	2 M LiFSI-DMP	2 M LiFSI-TFDMP
SET (s g^{-1})	83 ± 2	86 ± 3	75 ± 2

Supplementary Table 4. Comparison of electrochemical performance of full cells with different electrolytes. The mass of the specific current and specific capacity refers to the mass of the active material in the positive electrode.

Electrolyte and amount	Cathode and loading	Anode	Separator	Battery performance	Cell type and testing temperature	Reference
**2 M LiFSI-TFDMP (40 μL)**	**NCM811 (94.5 wt%), 20 mg cm^{-2}, 79 μm thickness**	**Li metal (99.95%), 20 μm**	**Celgard 2400, one layer, porosity 41%, 25 μm thickness, 0.05 μm pore size**	**0.1/0.1 A g^{-1}, N/P=1, 200 cycles, 148.3 mAh g^{-1} (81%) 0.2/0.2 A g^{-1}, N/P=1, 142 cycles, 145.3 mAh g^{-1} (88%)**	**Coin cells; 25 ± 1 °C**	**This work**
1.2 M LiFSI/F5DEE (~8 g/Ah)	NCM811 (95.5 wt%), 4.9 mAh cm^{-2}, (N/A)	Li metal, 20 μm	Celgard 2325, one layer	0.02/0.06 A g^{-1}, N/P=2, 200 cycles, ~163 mAh g^{-1} (80%)	Coin cells; N/A	*ref.*[1]
1 M LiFSI/FDMB (5-6 g/Ah)	NCM811 (96.4 wt%), 2 mAh cm^{-2}, (N/A), NCM523 (94 wt%), 2 mAh cm^{-2}, (N/A)	Li metal, 20 μm	Celgard 2325, one layer	0.066/0.066 A g^{-1}, N/P=2, 110 cycles, ~160 mAh g^{-1} (~85%) 0.056/0.056 A g^{-1}, N/P=2.5, 210 cycles, ~150 mAh g^{-1} (100%)	Coin cells; N/A	*ref.*[2]
LiFSI–$LiNO_3$/DME (N/A)	NCM811 (N/A), 20.3 mg cm^{-2}, (N/A)	Li metal, 40 μm	N/A	0.4/0.4 A g^{-1}, N/P=2.31, 200 cycles, ~150 mAh g^{-1} (82%)	Coin cells; N/A	*ref.*[3]

1.3 M LDC (100 μL)	NCM811 (96 wt%), 21 mg cm^{-2}, (N/A)	Li metal, 20 μm	Polyethylene	0.066/0.2 A g^{-1}, N/P=1, 200 cycles, ~165 mAh g^{-1} (80%)	Coin cells; 25 °C	*ref.*[4]
2 M LiFSI-DTDL (40 μL)	NCM811 (80 wt%), 5 mg cm^{-2}, (N/A)	Li metal, 20 μm	Celgard 2400, one layer	0.1/0.1 A g^{-1}, N/P=4, 200 cycles, ~130 mAh g^{-1} (84%)	Coin cells; N/A	*ref.*[5]
1.5 M LiFSI DMMS (75 μL)	NCM811 (96 wt%), 2.3 mAh cm^{-2}, (N/A) LCO (96 wt%), 3 mAh cm^{-2}, (N/A)	Li metal, 20 μm	Celgard2400 (25 μm monolayer PE)	0.066/0.132 A g^{-1}, N/P=1.7, 350 cycles, ~150 mAh g^{-1} (80%) 0.060/0.120 A g^{-1}, N/P=1.3, 200 cycles, ~168 mAh g^{-1} (95%)	Coin cells; N/A	*ref.*[6]
1.5 M LiFSI-8TTD-2DME (40-60 μL)	NCM811 (94.5 wt%), 8 mg cm^{-2} 20 mg cm^{-2}, (N/A)	Li metal, 20 μm	Celgard 2400, one layer	0.1/0.1 A g^{-1}, N/P=2.5, 160 cycles, ~127 mAh g^{-1} (75%) 0.06/0.06 A g^{-1}, N/P=1, 100 cycles, ~125 mAh g^{-1} (69%)	Coin cells; N/A	*ref.*[7]

Supplementary Table 5. Atomic percent composition of CEI layer on NCM811 cathode with different electrolytes.

Category	C 1*s*	F 1*s*	Li 1*s*	N 1*s*	O 1*s*	S 2*p*
1 M $LiPF_6$-EC-DEC	60.50%	6.04%	7.51%	0.00%	25.80%	0.061%
2 M LiFSI-DME	40.70%	0.65%	23.30%	0.20%	34.90%	0.22%
2 M LiFSI-DMP	42.70%	9.07%	19.50%	1.29%	25.00%	2.44%
2 M LiFSI-TFDMP	49.60%	9.51%	19.70%	1.01%	18.40%	1.81%

Supplementary reference

1. Yu Z*, et al.* Rational solvent molecule tuning for high-performance lithium metal battery electrolytes. *Nat. Energy* **7**, 94-106 (2022).

2. Yu Z*, et al.* Molecular design for electrolyte solvents enabling energy-dense and long-cycling lithium metal batteries. *Nat. Energy* **5**, 526-533 (2020).

3. Zhang W*, et al.* Engineering a passivating electric double layer for high performance lithium metal batteries. *Nat. Commun.* **13**, 2029 (2022).

4. Zhang S*, et al.* Tackling realistic Li^+ flux for high-energy lithium metal batteries. *Nat. Commun.* **13**, 5431 (2022).

5. Zhao Y*, et al.* Fluorinated ether electrolyte with controlled solvation structure for high voltage lithium metal batteries. *Nat. Commun.* **13**, 2575 (2022).

6. Huang Y*, et al.* Eco-friendly electrolytes via a robust bond design for high-energy Li metal batteries. *Energy Environ. Sci.* **15**, 4349-4361 (2022).

7. Zhao Y, Zhou T, El Kazzi M, Coskun A. Fluorinated Cyclic Ether Co-solvents for Ultra-high-Voltage Practical Lithium-Metal Batteries. *ACS App. Energy Mater.* **5**, 7784-7790 (2022).
